# Supplementary material for: Testing the heat dissipation limitation hypothesis: basal metabolic rates of endotherms decrease with increasing upper and lower critical temperatures
Source: PeerJ. 2018 Oct 31;6:e5725. doi: 10.7717/peerj.5725 (PMC6215442; doi:10.7717/peerj.5725)
Supplement: Supplemental Information 1 [file peerj-06-5725-s001.pdf]

References:

Bartholomew, George A & Macmillen, R.E. (1961) Water Economy of the California Quail and Its Use of Sea Water. *The Auk*, **78**, 505–514.

Bartholomew, G.A., Leitner, P. & Nelson, J.E. (1964) Body temperature, oxygeb consumption, and heart rate in three species of Australian flying foxes. *Physiological Zoology*, **37**, 179–198.

Baudinette, R. V. (1972) Energy metabolism and evaporative water loss in the California ground squirrel. *Journal of Comparative Physiology*, **81**, 57–72.

Baudinette, R. V, Churchill, S.K., Christian, K.A., Nelson, J.E. & Hudson, P.J. (2000) Energy, water balance and the roost microenvironment in three Australian cave-dwelling bats (Microchiroptera). *Journal of Comparative Physiology. B, Biochemical, Systemic, and Environmental Physiology*, **170**, 439–46.

Bell, G.P., Bartholomew, G.A. & Nagy, K.A. (1986) The roles of energetics, water economy, foraging behavior, and geothermal refugia in the distribution of the bat, *Macrotus californicus*. *Journal of Comparative Physiology B: Biochemical, Systemic, and Environmental Physiology*, **156**, 441–450.

Ben-Hamo, M., Pinshow, B., McCue, M.D., McWilliams, S.R. & Bauchinger, U. (2010) Fasting triggers hypothermia, and ambient temperature modulates its depth in Japanese quail *Coturnix japonica*. *Comparative Biochemistry and Physiology Part A: Molecular & Integrative Physiology*, **156**, 84–91.

Booth, D.T. (1989) Metabolism in malleefowl (*Leipoa ocellata*). *Comparative Biochemistry and Physiology A: Molecular & Integrative Physiology*, **92A**, 207–209.

Bozinovic, F. & Rosenmann, M. (1988a) Comparative energetics of South American cricetid rodents. *Comparative Biochemistry and Physiology Part A: Molecular & Integrative Physiology*, **91**, 195–202.

Bozinovic, F. & Rosenmann, M. (1988b) Daily torpor in *Calomys musculinus*, a South American rodent. *Journal of Mammalogy*, **69**, 150–152.

Bozinovic, F., Ruiz, G., Cortes, A. & Rosenmann, M. (2005) Energetics , thermoregulation and torpor in the Chilean mouse-opossum *Thylamys elegans* (Didelphidae). *Revista Chilena de Historia Natural*, **78**, 199–206.

Bradley, S.R. & Hudson, J.W. (1974) Temperature regulation in the tree shrew *Tupaia glis*. *Comparative Biochemistry and Physiology Part A: Molecular & Integrative Physiology*, **48**, 55–60.

Bradley, W.G., Miller, J.S. & Yousef, M.K. (1974) Thermoregulatory patterns in pocket gophers : desert and mountain. *Physiological Zoology*, **47**, 172–179.

Bradley, W.G. & Yousaf, M.K. (1975) Thermoregulatory responses in the plains pocket gopher, *Geomys bursarius*. *Comparative Biochemistry and Physiology A: Molecular & Integrative Physiology*, **52**, 35–38.

Bradley, W.G., Yousef, M.K. & Scott, I.M. (1975) Physiological studies on the rock pocket mouse, Perognathus intermedius. *Comparative Biochemistry and Physiology Part A: Molecular & Integrative Physiology*, **50A**, 331–337.

Brent, R., Pedersen, P.F., Bech, C. & Johansen, K. (1985) Thermal balance in the European Coot *Fulica atra* exposed to temperatures from -28 C to 40 C. *Ornis Scandinavia*, **16**, 145–150.

Breyen, L.J., Bradely, W.G. & Yousef, M.K. (1973) Physiological and ecological studies on the chisel-

toothed kangroos rat, *Dipodomys microps*. *Comparative Biochemistry and Physiology A: Molecular & Integrative Physiology*, **44**, 543–555.

Buffenstein, R. (1984) The importnace of microhabitat in thermoregulation and thermal conductance in two Namib rodents- a crevice dweller, *Aethomys namaquensis* and a burrow dweller, *Gerbillurus paeba*. *Journal of Thermal Biology*, **9**, 235–241.

Buffenstein, R. & Jarvis, J.U.M. (1985) Thermoregulation and metablism in the smallest African gerbil, *Gerbillus pusillus*. *Journal of Zoology*, **205**, 107–121.

Calder, W.A. & Schmidt-Nielsen, K. (1967) Temperature evaporation regulation and in the pigeon and the roadrunner. *American Journal of Physiology*, **213**, 883–889.

Collins, B.G. & Bradshaw, S.D. (1973) Studies on the metabolism, thermoregulation, and evporative water losses of two species of Australian rats, *Rattus villosissimus* and *Rattus rattus*. *Physiological Zoology*, **46**, 1–21.

Coulombe, H.N. (1970) Physiological and physical aspects of temperature regulation in the burrowing owl *Speotyto cunicularia*. *Comparative Biochemistry and Physiology*, **35**, 307–337.

Cruz-Neto, A.P. & Abe, A.S. (1997) Taxa metabolica e termorregulacao no morcego nectarivoro, *Glossophaga soricina* (Chiroptera, phyllostomatidae). *Revista Brasileira de Biologia*, **57**, 203–209.

Cryan, P.M. & Wolf, B.O. (2003) Sex differences in the thermoregulation and evaporative water loss of a heterothermic bat, *Lasiurus cinereus*, during its spring migration. *Journal of Experimental Biology*, **206**, 3381–3390.

Dawson, W.R. (1955) The relation of oxygen consumption to temperature in desert rodents. *Journal of Mammalogy*, **36**, 543–553.

Dawson, W.R. & Bennett, A.F. (1973) Roles of metabolic level and temperature regulation in the adjustment of western plumed pigeons (*Lophophaps ferruginea*) to desert conditions. *Comparative Biochemistry and Physiology A: Molecular & Integrative Physiology*, **44**, 249–66.

Dawson, W.R. & Bennett, A.F. (1978) Energy metabolism and thermoregulation of the spectacled hare wallaby (*Lagorchestes conspicillatus*). *Physiological Zoology*, **51**, 114–130.

Dawson, W.R. & Fisher, C.D. (1969) Responses to temperature by the spotted nightjar (*Eurostopodus guttatus*). *The Condor*, **71**, 49–53.

Dawson, W.R. & Fisher, C.D. (1982) Observations on the temperature regulation and water economy of the galah (*Cacatua roseicapilla*). *Comparative Biochemistry and Physiology A: Molecular & Integrative Physiology*, **72**, 1–10.

Dawson, T.J. & Hulbert, A.J. (1970) Standard metabolism, body temperature, and surface areas of Australian marsupials. *The American Journal of Physiology*, **218**, 1233–1238.

Dawson, T. & Schmidt-Nielsen, K. (1966) Effect of thermal conductance on water economy in the antelope jack rabbit, *Lepus alleni*. *Journal of Cellular Physiology*, **67**, 463–472.

Degabriele, R. & Dawson, T.J. (1979) Metabolism and heat balance in an arboreal marsupial, the koala (Phascolarctos cinereus). *Journal of Comparative Physiology B: Biochemical, Systemic, and Environmental Physiology*, **134**, 293–301.

Degen, A.A., Pinshow, B., Yosef, R., Kam, M. & Nagy, K.A. (1992) Energetics and growth rate of Northern shrike (Lanius Excubitor) nestlings. *Ecology*, **73**, 2273–2283.

Dempster, E.R., Perrin, M.R. & Downs, C.T. (1999) *Gerbillurus vallinus*. *Mammalian Species*, **605**, 1–4.

Dempster, E.R., Perrin, M.R., Downs, C.T. & Griffin, M. (1998) *Gerbillurus setzeri*. *Mammalian Species*, **598**, 1–4.

Downs, C.T. & Perrin, M.R. (1990) Thermal parameters of four species of *Gerbillurus*. *Journal of Thermal Biology,* **15**, 291–300.

Downs, C.T. & Perrin, M.R. (1995) The thermal biology of three southern African elephant-shrews. *Journal of Thermal Biology*, **20**, 445–450.

Edwards Jr., T.C. (1987) Standard rate of metabolism in the common barn-owl (*Tyto alba*). *The Wilson Bulletin*, **99**, 704–706.

Ehlers, R. & Morton, M.L. (1982) Metabolic rate and evaporative water loss in the least seed-snipe, *Thinocorus rumicivorus*. *Comparative Biochemistry and Physiology Part A: Molecular & Integrative Physiology,* **73**, 233–235.

Fleming, M.R. (1980) Thermoregulation and torpor in the sugar glider, *Petaurus breviceps* (Marsupialia: Petauridae). *Australian Journal of Zoology,* **28**, 521–534.

Flint, E.N. & Nagy, K.A. (1984) Flight energetics of free-living sooty terns. *The Auk*, **101**, 288–294.

Ganey, J.L., Balda, R.P. & King, R.M. (1993) Metabolic rate and evaporative water loss of Mexican spotted and great horned owls. *Wilson Bull*, **105**, 645–656.

Geiser, F. (1987) Hibernation and daily torpor in two pygmy possum (*Cercartetus* spp., Marsupialia). *Physiological Zoology*, **60**, 93–102.

Golightly Jr., R.T. & Ohmart, R.D. (1983) Metabolism and body temperature of two desert canids: coyotes and kit foxes. *Journal of Mammalogy,* **64**, 624–635.

Haim, A., Aarde, R.J. Van & Skinner, J.D. (1990) Metabolism and thermoregulation in the Cape porcupine, *Hystrix africaeaustralis*. *Physiological Zoology*, **63**, 795–802.

Haim, A. & Fairall, N. (1987) Bioenergetics of an herbivorous rodent *Otomys irroratus*. *Physiological Zoology,* **60**, 305–309.

Hänßler, I. (1978) *Der Einfluss Des Sexualhormons "Testosteron" auf Die Körpertemperatur Und Den Stoffwechsel Bei Der Wachtel (Coturnix Coturnix Japonica)*. J.W.Goethe University, Frankfurt.

Hayworth, A.M. & Weathers, W.W. (1984) Temperature regulation and climate adaptation in black-billed and yellow-billed magpies. *The Condor*, **86**, 19–26.

Hinds, D.S. (1973) Acclimatization of thermoregulation in the desert cottontail, *Sylvilagus audubonii*. *Journal of Mammalogy*, **54**, 708–728.

Hinds, D.S. & Calder, W.A. (1973) Temperature regulation of the *Phyrrhuloxia* and the Arizona Cardinal. *Physiological Zoology,* **46**, 55–71.

Hinsley, S.A., Ferns, P.N., Thomas, D.H. & Pinshow, B. (1993) Black-bellied sandgrouse (*Pterocles orientalis*) and Pin-tailed sandgrouse (*Pterocles alchata*): closely related species with differing bioenergetic adaptations to arid zones. *Physiological Zoology,* **66**, 20–42.

Hooper, E.T. & Hilali, M. El. (1972) Temperature regulation and habits in two species of jerboa, Genus *Jaculus*. *Journal of Mammalogy*, **53**, 574–593.

Hudson, J.W. & Brush, A.H. (1964) A comparative study of the cardiac and metabolic performance of the

dove, *Zenaidura macrorura*, and the quail, *Lophortyx californicus*. *Comparative Biochemistry and Physiology*, **12**, 157–170.

Hudson, J.W. & Kimzey, S.L. (1966) Temperature regulation and metabolic rhythms in populations of the house sparrow, *Passer domesticus*. *Comparative biochemistry and physiology*, **17**, 203–17.

Jin-Song, L., De-Hua, W. & Ru-Yong, S. (2005) Climatic adaptations in metabolism of four species of small birds in China. *Acta Zoologica Sinica*, **1**, 24–30.

Jin-Song, L., De-Hua, W., Ying, W., Ming-Huan, C., Chun-Guang, S. & Ru-Yong, S. (2004) Energetics and thermoregulation of the *Carpodacus roseus, Fringlla montifringilla* and *Acanthis flammea*. *Acta Zoologica Sinica*, **50**, 357–363.

Jin-Song, L., Zhi-Yan, Z., Hong, M. & Zeng-Suan, H. (2001) Characterstics of resting metabolic rate in little bunting (*Emberiza pusilla*) and chestnut bunting (*E. rutila*). *Acta Zoologica Sinica*, **47**, 347–350.

Johnson, R.E. (1968) Temperature regulation in the white-tailed ptarmigan, *Lagopus leucurus*. *Comparative Biochemistry and Physiology*, **24**, 1003–14.

Kennedy, P.M. & Macfarlane, W. V. (1971) Oxygen consumption and water turnover of the fat-tailed marsupials *Dasycercus cristicauda* and *Sminthopsis crassicaudata*. *Comparative Biochemistry and Physiology Part A: Molecular & Integrative Physiology*, **40A**, 723–732.

Kinnear, A. & Shield, J.W. (1975) Metabolism and temperature regulation in marsupials. *Comparative Biochemistry and Physiology Part A: Molecular & Integrative Physiology*, **52A**, 235–45.

Knudsen, K.L. & Kilgore Jr., D.L. (1990) Temperature regulation and basal metabolic rate in the spotted skunk, *Spilogale putorius*. *Comparative Biochemistry and Physiology Part A: Molecular & Integrative Physiology*, **97A**, 27–33.

Król, E. (1994) Metabolism and thermoregulation in the eastern hedgehog *Erinaceus concolor*. *Journal of Comparative Physiology B: Biochemical, Systemic, and Environmental Physiology*, **164**, 503–507.

Lane, J.E., Swanson, D.L., Brigham, R.M. & McKechnie, A.E. (2004) Physiological responses to temperature by whip-poor-wills : more evidence for the evolution of low metabolic rates in Caprimulgiformes. *The Condor*, **106**, 921–925.

Lasiewski, R.C. & Dawson, W.R. (1964) Physiological responses to temperature in the common nighthawk. *The Condor*, **66**, 477–490.

Lasiewski, R.C., Dawson, W.R. & Bartholomew, G.A. (1970) Temperature regulation in the little Paupan frogmouth, *Podargus ocellatus*. *The Condor*, **72**, 332–338.

Le-Maho, Y., Goffart, M., Rochas, A., Felbabel, H. & Chatonnet, J. (1981) Thermoregulation in the only nocturnal simian, the night monkey *Aotus trivirgatus*. *American Journal of Physiology*, **240**, 156–165.

Leitner, P. & Nelson, J.E. (1967) Body temperature, oxygen consumption and heart rate in the Australian false vampire bat, *Macroderma gigas*. *Comparative Biochemistry and Physiology*, **21**, 65–74.

Leon, B., Shkolnik, A. & Shkolnik, T. (1983) Temperature regulation and water metabolism in the elephant shrew *Elephantulus edwardi*. *Comparative Biochemistry and Physiology Part A: Molecular & Integrative Physiology*, **74**, 399–407.

Ligon, J.D. (1968) *The Biology of the Elf Owl , Micrathene Whitneyi*.

Ligon, J.D. (1969) Some aspects of temperature relations in small Owls. *The Auk*, **86**, 458–472.

Lill, A., Box, J. & Baldwin, J. (2006) Do metabolism and contour plumage insulation vary in response to seasonal energy bottlenecks in superb fairy-wrens? *Australian Journal of Zoology*, **54**, 23–30.

Lovegrove, B.G. (1986) The metabolism of social subterranean rodents: adaptation to aridity. *Oecologia*, **69**, 551–555.

Lovegrove, B.G. (1987) Thermoregulation in the subterranean rodent *Georychus capensis* (Rodentia: Bathyergidae). *Physiological Zoology*, **60**, 174–180.

Lovegrove, B.G., Heldmair, G. & Knight, M. (1991) Seasonal and circadian energetic patterns in an arboreal rodent, *Thallomys paedulcus*, and a burrow-dwelling rodent, *Aethomys namaquensis*, from the Kalahari desert. *Journal of Thermal Biology*, **4**, 199–209.

MacMillen, R.E. & Lee, A.K. (1970) Energy metabolism and pulmocutaneous water loss of Australian hopping mice. *Comparative Biochemistry and Physiology*, **35**, 355–369.

MacMillen, R.E., Whittow, G.C., Christopher, E.A. & Ebisu, R.J. (1977) Oxygen consumption, evaporative water loss, and body temperature in the sooty terns. *The Auk*, **94**, 72–79.

Marder, J. & Bernstein, R. (1983) Heat balance of the partridge *Alectoris chukar* exposed to moderate, high and extreme thermal stress. *Comparative biochemistry and physiology Part A: Molecular & Integrative Physiology*, **74**, 149–54.

McNab, B.K. (1969) The economics of temperature regulation in neutropical bats. *Comparative Biochemistry and Physiology*, **31**, 227–268.

McNab, B.K. (1979) Climatic adaptation in the energetics of *heteromyid* rodents. *Comparative Biochemistry and Physiology A: Molecular & Integrative Physiology*, **62A**, 813–820.

McNab, B.K. (1980) Food habits, energetics, and the population biology of mammals. *The American Naturalist*, **116**, 106–124.

McNab, B.K. (1989) Temperature regulation and rate of metabolism in three Bornean bats. *Journal of Mammalogy*, **70**, 153–161.

McNab, B.K. (2000) The influence of body mass, climate, and distribution on the energetics of South Pacific pigeons. *Comparative Biochemistry and Physiology. Part A, Molecular & Integrative Physiology*, **127**, 309–29.

McNab, B.K. (2003) The energetics of New Zealand's ducks. *Comparative biochemistry and physiology Part A: Molecular & Integrative Physiology*, **135**, 229–247.

McNab, B.K. & Bonaccorso, F.J. (1995) The energetics of Australasian swifts, frogmouths, and nightjars. *Physiological Zoology*, **68**, 245–261.

McNab, B.K. & Morrison, P. (1963) Body temperature and metabolism in subspecies of Peromyscus from arid and mesic environments. *Ecological Monographs*, **33**, 63–82.

Merola-Zwartjes, M. & Ligon, J.D. (2000) Ecological energetics of the Puerto Rican tody: heterothermy, torpor, and intra-island variation. *Ecology*, **81**, 990–1003.

Ming, L., Jin-Song, L., Hong-lei, H., Hai-jun, Z. & Hao, F. (2005) Metabolism and thermoregulation in waxwings (*Bombycilla garrulous*) and black-faced buntings (*Emberiza spodocephala*). *Zoological Research*, **26**, 287–293.

Moldenhauer, R.R. (1970) The effects of temperature on the metabolic rate and evaporative water loss of the sage sparrow *Amphispiza belli nevadensis*. *Comparative Biochemistry and Physiology*, **36**, 579–587.

Mugaas, J.N. & Templeton, J.R. (1970) Thermoregulation in the red-breasted nuthatch (*Sitta canadensis*). *The Condor*, **72**, 125–132.

Mugas, J.N., Seidensticker, J. & Mahlke-Johnson, K.P. (1993) *Metabolic Adaptation to Climate and Distribution of the Racoon Procyon Lotor and Other Protocyonidae*.

Müller, E.F. (1979) Energy metabolism, thermoregulation and water budget in the slow loris (Nycticebus coucang, Boddaert 1785). *Comparative Biochemistry and Physiology A: Molecular & Integrative Physiology*, **64**, 109–119.

Müller, E.F., Kamau, J.M.Z. & Maloiy, G.M.O. (1983) A comparative study of basal metabolism and thermoregulation in a folivorous (*Colobus guereza*) and an omnivorous (*Cercopithecus mitis*) primate species. *Comparative Biochemistry and Physiology Part A: Molecular & Integrative Physiology*, **74**, 319–322.

Müller, E.F. & Lojewski, U. (1986) Thermoregulation in the meerkat (*Suricata suricatta* schreber, 1776). *Comparative Biochemistry and Physiology Part A: Molecular & Integrative Physiology*, **83**, 217–224.

Nelson, Z.C. & Yousef, M.K. (1979) Thermoregulatory responses of desert wood rats, *Neotoma lepida*. *Comparative Biochemistry and Physiology A: Molecular & Integrative Physiology*, **63**, 109–113.

Noll-Banholzer, U. (1979) Body temperature, oxygen consumption, evaportaive water loss and heart rate in the fennec. *Comparative Biochemistry and Physiology A: Molecular & Integrative Physiology*, **62**, 585–592.

Reinking, L.N., Kilgore Jr., D.L., Fairbanks, E.S. & Hamilton, J.D. (1977) Temperature regulation in normothermic black-tailed prairie dogs, *Cynomys ludovicianus*. *Comparative Biochemistry and Physiology A: Molecular & Integrative Physiology*, **57**, 161–165.

Rising, J.D. (1969) A comparison of metabolism and evaporative water loss of Baltimore and bullock orioles. *Comparative Biochemistry and Physiology*, **31**, 915–925.

Rodríguez-Durán, A. & Rodriguez-Duran, A. (1995) Metabolic rates and thermal conductance in four species of neotropical bats roosting in hot caves. *Comparative Biochemistry and Physiology Part A: Molecular & Integrative Physiology*, **110**, 347–355.

Salt, G.W. (1952) The relation of metabolism to climate and distribution in three finches of the genus *Carpodacus*. *Ecological Monographs*, **22**, 121–152.

Soriano, P.J., Ruiz, A. & Arends, A. (2002) Physiological responses to ambient temperature manipulation by three species of bats from Andean cloud forests. *Journal of Mammalogy*, **83**, 445–457.

Sparti, A. (1990) Comparative temperature regulation of African and European shrews. *Comparative Biochemistry and Physiology Part A: Molecular & Integrative Physiology*, **97A**, 391–397.

Tieleman, B.I. & Williams, J.B. (2002) Cutaneous and respiratory water loss in larks from arid and mesic environments. *Physiological and Biochemical Zoology*, **75**, 590–9.

Trost, C.H. (1972) Adaptations of horned larks (*Eremophila alpestris*) to hot environments. *The Auk*, **89**, 506–527.

Vleck, C.M.. & Vleck, D. (1979) Metabolic rate in five tropical bird species. *The Condor*, **81**, 89–91.

Wang, L.C.-H. & Hudson, J.W. (1971) Temperature regulation in normothermic and hibernating eastern chipmunk, *Tamias striatus*. *Comparative Biochemistry and Physiology Part A: Molecular & Integrative Physiology*, **38A**, 59–90.

Ward, D. & Pinshow, B. (1995) Temperature regulation of the great grey shrike (*Lanius excubitor*) in the Negev desert—I. Laboratory measurements of metabolic rate and evaporative water loss. *Journal of Thermal Biology*, **20**, 263–269.

Weathers, W.W. (1977) Temperature regulation in the dusky munia, *Lonchura fuscans* (Cassin) (Estrildidae). *Australian Journal of Zoology*, **25**, 193–199.

Weathers, W.W. (1981) Physiological thermoregulation in heat-stressed birds: consequences of body size. *Physiological Zoology*, **54**, 345–361.

Weathers, W.W. & Caccamise, D.F. (1975) Temperature regulation and water requirements of the monk parakeet , *Myiopsitta monachus*. *Oecologia*, **18**, 329–342.

Weathers, W.W. & Schoenbaechler, D.C. (1976) Regulation of body temperature in the budgerygah, *Melopsittacus undulatus*. *Australian Journal of Zoology*, **24**, 39–47.

Weathers, W.W., Shapiro, C.J. & Astheimer, B. (1980) Metabolic responses of cassin's finches (*Carpodacus cassinii*) to temperature. *Comparative Biochemistry and Physiology A: Molecular & Integrative Physiology*, **65A**, 235–238.

Wells, R.T. (1978) Thermoregulation and activity rhythms in the hairy-nosed wombat, *Lasiorhinus latifrons* (Owen), (Vombatidae). *Australian Journal of Zoology*, **26**, 639–651.

Whiteford, W.G. & Conley, M.I. (1971) Oxygen consumption and water metabolism in a carnivorous mouse. *Comparative Biochemistry and Physiology A: Molecular & Integrative Physiology*, **40A**, 797–803.

Whittow, G.C., Gould, E. & Rand, D. (1977a) Body temperature, oxygen consumption, and evaporative water loss in a primitive insectivore, the moon rat, *Echinosorex gymnurus*. *Journal of Mammalogy*, **58**, 233–235.

Whittow, G.C., Scammell, C.A., Leong, M. & Rand, D. (1977b) Temperature regulation in the smallest ungulate, the lesser mouse deer (Tragulus javanicus). *Comparative Biochemistry and Physiology A: Molecular & Integrative Physiology*, **56**, 23–26.

Willems, N.J. & Armitage, K.B. (1975) Thermoregulation and water requirements in semiarid and montane populations of the least chipmunk, *Eutamias minimus*-II. Water balance. *Comparative Biochemistry and Physiology A: Molecular & Integrative Physiology*, **52A**, 109–20.

Williams, J.B. (1999) Heat production and evaporative water loss of Dune larks from the Namib desert. *The Condor*, **101**, 432–438.

Ying, L., Tai-lin, Y., Cheng-ming, H., Tong, Z., Han-hua, L. & Chang-jian, L. (2011) Metabolism and thermoregulation between Mrs Hume's Pheasant (*Syrmaticus humiae*) and Elliot's Pheasant (*S. ellioti*). *Zoological Research*, **32**, 396–402.

Zervanos, S.M. (1975) Seasonal effects of temperature on the respiratory metabolism of the collared peccary (*Tayassu tajacu*). *Comparative Biochemistry and Physiology A: Molecular & Integrative Physiology*, **50A**, 365–371.

Zhang, L., Cai, J. & Wang, Z. (2012) Metabolism and thermoregulation in the tree shrew, *Tupaia belangeri*. *Journal of Stress Physiology and Biochemistry*, **8**, 167–178.
