# Supplementary material for: Testing the heat dissipation limitation hypothesis: basal metabolic rates of endotherms decrease with increasing upper and lower critical temperatures
Source: PeerJ. 2018 Oct 31;6:e5725. doi: 10.7717/peerj.5725 (PMC6215442; doi:10.7717/peerj.5725)
Supplement: Supplemental Information 2 [file peerj-06-5725-s002.pdf]

Supplementary table S1

Species	Order	Family	Taxa	Mass (g)	BMR (KJ/h)	Tlc (°C)	Tuc (°C)	Reference
Abrothrix andinus	Rodentia	Muridae	Mammal	34.6	1.3	26.8	34	Bozinovic & Rosenmann, 1988
Abrothrix longipilis	Rodentia	Muridae	Mammal	42.3	1.16	27.3	32	Bozinovic & Rosenmann, 1988
Ammospermophilus leucurus	Rodentia	Sciuridae	Mammal	96	1.99	31	34	Dawson, 1955
Aotus trivirgatus	Primates	Aotidae	Mammal	1020	9.22	28	30	Le-Maho *et al*., 1981
Artibeus jamaicensis	Chiroptera	Phyllostomidae	Mammal	45.2	1.14	25	35	McNab, 1969
Artibeus lituratus	Chiroptera	Phyllostomidae	Mammal	70.1	1.74	25	36	McNab, 1969
Bettongia gaimardi	Diprutodontia	Potoroidae	Mammal	1700	12.88	10	20	Rose, 1997
Cabassous centralis	Rodentia	Dasypodidae	Mammal	3810	16.07	27.5	32.5	McNab, 1980
Canis latrans	Carnivora	Canidae	Mammal	10000	54	22	26	Golightly *et al*., 1983
Cannomys badius	Rodentia	Muridae	Mammal	344	3.45	26.74	34.5	McNab, 1979
Carollia perspicillata	Chiroptera	Phyllostomidae	Mammal	14.9	0.63	29	35	McNab, 1969
Cercartetus lepidus	Diprutodontia	Burramyidae	Mammal	12	0.38	29	33	Geiser, 1987
Cercartetus nanus	Diprutodontia	Pseudocheiridae	Mammal	70	1.21	31	35	Bartholomew & MacMillen, 1961
Cercopithecus mitis	Diprutodontia	Pseudocheiridae	Mammal	8500	68.27	5	28	Muller *et al*., 1983
Chaetodipus intermedius	Rodentia	Heteromyidae	Mammal	15.2	0.31	33	36	Bradley *et al*., 1975
Colobus guereza	Primates	Cercopithecidae	Mammal	10500	60.09	5	28	Muller *et al*., 1983
Crocidura russula	Eulipotyphla	Soricidae	Mammal	10.4	0.46	27.5	35	Sparti, 1990
Crocidura suaveolens	Eulipotyphla	Soricidae	Mammal	6.5	0.38	27.5	35	Sparti, 1990
Cryptomys damarensis	Rodentia	Bathyergidae	Mammal	124	1.43	27	31	Lovegrove, 1986
Cynomys ludovicianus	Rodentia	Sciuridae	Mammal	1112.3	8.49	30.2	35	Reinking *et al*., 1977
Cynopterus brachyotis	Chiroptera	Pteropodidae	Mammal	37.4	0.95	30	37	McNab, 1989
Dasycercus cristicauda	Dasyuromorphia	Dasyuridae	Mammal	86	0.93	30	37	Kennedy & Macfarlane, 1971
Diphylla ecaudata	Chiroptera	Phyllostomidae	Mammal	27.8	0.68	26	30	McNab, 1969
Dipodomys microps	Rodentia	Heteromyidae	Mammal	57.2	1.34	27	32	Breyen *et al*., 1973
Elephantulus edwardii	Macroscelidea	Macroscelididae	Mammal	49.8	1.09	32.5	36	Leon *et al*., 1983
Erinaceus concolor	Erinaceomorpha	Erinaceidae	Mammal	822.7	6.97	27.5	31.5	Krol, 1994
Georychus capensis	Rodentia	Bathyergidae	Mammal	192.6	2.26	26.3	34	Lovvegrove, 1987
Gerbillurus paeba	Rodentia	Muridae	Mammal	33.9	0.7	32.3	35.1	Buffenstein, 1984
Gerbillurus setzeri	Rodentia	Muridae	Mammal	46.1	0.74	32.2	34.8	Dempster *et al*., 1998
Gerbillurus tytonis	Rodentia	Muridae	Mammal	29.9	0.64	32.4	34.9	Downs & Perrin, 1990
Gerbillurus vallinus	Rodentia	Muridae	Mammal	38.8	0.7	33.1	35	Dempster *et al*., 1999
Gerbillus pusillus	Rodentia	Muridae	Mammal	12.6	0.27	31.4	38	Buffenstein & Jarvis, 1985
Glossophaga soricina	Chiroptera	Phyllostomidae	Mammal	9.6	0.43	31.4	35.2	Cruz-Neto & Abe, 1997
Hystrix africaeaustralis	Rodentia	Hystricidae	Mammal	10700	40.82	24	27	Haim *et al*., 1990
Jaculus jaculus	Rodentia	Dipodidae	Mammal	74.5	1.85	33	35	Hooper & Hilali, 1972
Jaculus orientalis	Rodentia	Dipodidae	Mammal	139.1	2.79	28	33	Hooper & Hilali, 1972
Lagorchestes conspicillatus	Diprutodontia	Macropodidae	Mammal	2660	17.09	25	35	Dawson & Bennet, 1978
Lasiorhinus latifrons	Diprutodontia	Vombatidae	Mammal	25000	50.2	25	39	Wells, 1978
Lasiurus cinereus	Chiroptera	Vespertilionidae	Mammal	27.5	0.33	30	34	Cryan & Wolf, 2003
Lepus alleni	Lagomorpha	Leporidae	Mammal	3362	30.45	25	35	Dawson & Schmidt-Nileson, 1966
Macroderma gigas	Chiroptera	Megadermatidae	Mammal	107	1.89	30	35	Baudinette *et al*., 2000; Leitner & Nelson, 1966
Macrotis lagotis	Peramelemorpha	Thylacomyidae	Mammal	1011	7.11	27	35	Kinnear & Shield, 1975
Megadontomys thomasi	Rodentia	Cricetidae	Mammal	111	2.49	28	35	Buffenstein & Jarvis, 1985
Microtus californicus	Rodentia	Cricetidae	Mammal	11.7	1.37	33	40	Bell *et al*., 1986
Microtus longicaudus	Rodentia	Cricetidae	Mammal	26.9	1.25	26.5	33.5	Bell *et al*., 1986
Miniopterus schreibersii	Chiroptera	Vespertilionidae	Mammal	10.91	0.52	32.5	37.5	Baudinette *et al*., 2000
Mystromys albicaudatus	Rodentia	Muridae	Mammal	93.78	2.54	20	30	Downs & Perrin, 1995
Nasua nasua	Carnivora	Procyonidae	Mammal	4000	20.08	25	33	Mugaas *et al*., 1993
Neotoma lepida	Rodentia	Cricetidae	Mammal	106	2	31	35	Nelson & Yousaf, 1979
Noctilio leporinus	Chiroptera	Noctilionidae	Mammal	61	0.94	28	38	McNab, 1969
Notomys alexis	Rodentia	Muridae	Mammal	32.3	0.84	32	34	MacMillen & Lee, 1970
Notomys cervinus	Rodentia	Muridae	Mammal	34.2	0.9	33	34	MacMillen & Lee, 1970
Nycticebus coucang	Primates	Lorisidae	Mammal	1300	6.27	25	33	Muller, 1979
Onychomys torridus	Rodentia	Cricetidae	Mammal	19.1	0.78	30	35	Whitford & Conley, 1971
Otomys irroratus	Rodentia	Muridae	Mammal	111.6	1.86	24	28	Haim & Fairall, 1987
Pecari tajacu	Artiodactyla	Tayassuidae	Mammal	20000	116.82	28	35	Zervanos, 1975
Peromyscus californicus	Rodentia	Cricetidae	Mammal	49.6	1.17	27	34.5	McNab & Morrison, 1963
Peromyscus crinitus	Rodentia	Cricetidae	Mammal	20.9	0.62	29	35	McNab & Morrison, 1963
Peromyscus truei	Rodentia	Cricetidae	Mammal	33	1.25	27	33	McNab & Morrison, 1963
Petauroides volans	Diprutodontia	Pseudocheiridae	Mammal	1141	11.45	18	25	Rübsamen *et al*., 1984
Petaurus breviceps	Diprutodontia	Petauridae	Mammal	128.1	1.78	27	31	Fleming, 1980; Dawson & Hulbert, 1970
Phascolarctos cinereus	Diprutodontia	Phascolarctidae	Mammal	4765	21.05	20	26	Degabriele & Dawson, 1979
Phyllostomus discolor	Chiroptera	Phyllostomidae	Mammal	33.5	0.69	25	37	McNab, 1969
Phyllostomus hastatus	Chiroptera	Phyllostomidae	Mammal	84.2	1.42	25	35	McNab, 1969
Phyllotis darwini	Rodentia	Cricetidae	Mammal	59	1.43	27.5	34	Bozinovic & Rosenmann, 1988
Potos flavus	Carnivora	Procyonidae	Mammal	2400	15.23	23	30	Muller *et al*., 1983
Procavia capensis	Hyracoidea	Hyracoidea	Mammal	2400	13.01	27	35	Rubsamen *et al*., 1979
Pseudocheirus peregrinus	Diprutodontia	Pseudocheiridae	Mammal	872	5.7	25	32.5	Kinnear & Shield, 1975
Pteronotus quadridens	Chiroptera	Mormoopidae	Mammal	4.9	0.12	30	38	Rodriguez-Duran, 1995
Pteropus scapulatus	Chiroptera	Pteropodidae	Mammal	440	4.87	24	35	Bartholomew *et al*., 1964
Rattus villosissimus	Rodentia	Muridae	Mammal	250.6	2.92	30	35	Collins & Brdshaw, 1973
Setonix brachyurus	Diprutodontia	Macropodidae	Mammal	2674	16.13	20	32.5	Kinnear & Shield, 1975
Spermophilus beecheyi	Rodentia	Sciuridae	Mammal	599.6	6.38	25	30	Baudinette, 1972
Spilogale putorius	Carnivora	Mephitidae	Mammal	624	5.89	30	36	Knudsen & Kilgore, 1990
Sturnira erythromos	Chiroptera	Phyllostomidae	Mammal	15.9	0.8	25.5	32.5	Soriano *et al*., 2002
Suricata suricatta	Carnivora	Herpestidae	Mammal	850	6.32	30	33	Muller & Lojewski, 1986
Sylvilagus audubonii	Lagomorpha	Leporidae	Mammal	672.4	8.79	28	40	Hinds, 1973
Tamias minimus	Rodentia	Sciuridae	Mammal	50.6	1.54	26	34	Willems & Armitage, 1975
Tamias striatus	Rodentia	Sciuridae	Mammal	92.2	1.81	28.5	32	Wang & Hudson, 1971
Thallomys paedulcus	Rodentia	Muridae	Mammal	124.7	1.38	27.46	35.89	Lovegrove *et al*., 1991
Thomomys bottae	Rodentia	Geomyidae	Mammal	143	2.41	28	30	Vleck, 1979
Thomomys talpoides	Rodentia	Geomyidae	Mammal	106	2.19	26	32	Bradley *et al*., 1974
Thomomys umbrinus	Rodentia	Geomyidae	Mammal	85	1.45	27	35	Bradley *et al*., 1974
Thylamys elegans	Didelphimorpha	Didelphidae	Mammal	40.2	0.86	27.5	35	Bozinovic *et al*., 2005
Tragulus javanicus	Artiodactyla	Tragulidae	Mammal	1618	17.59	26.6	29	Whittow *et al*.,1977
Tupaia belangeri	Scandentia	Tupaiidae	Mammal	186	2.54	27.5	35	Zhang *et al*., 2012
Vulpes macrotis	Carnivora	Canidae	Mammal	1868	18.83	22	33	Golightly & Omhart, 1983
Vulpes zerda	Carnivora	Canidae	Mammal	1106	8	23.4	32	Noll-Banholzer, 1979
Aegolius acadicus	Strigiformes	Strigidae	Bird	118.1	2.45	21	33	Ligon, 1969
Alaemon alaudipes	Passeriformes	Alaudidae	Bird	37.7	1.54	32.7	37.5	Tieleman & Williams, 2002
Alectoris chukar	Galliformes	Phasianidae	Bird	475	7.06	24	39	Marder & Bernstein, 1983
Amphispiza belli	Passeriformes	Emberizidae	Bird	18.6	1.29	28	37	Moldenhauer, 1970
Athene cunicularia	Passeriformes	Strigidae	Bird	146.7	2.8	25	37	Coulombe, 1970
Bubo virginianus	Strigiformes	Strigidae	Bird	1000	15.62	20.3	32.2	Ganey *et al*., 1993

Cacatua roseicapilla	Psittaciformes	Cacatuidae	Bird	271	5.04	22	33	Dawson & Fisher, 1982
Callipepla gambelii	Galliformes	Odontophoridae	Bird	125.5	2.72	34	42	Weathers, 1981
Caprimulgus vociferus	Caprimulgiformes	Caprimulgidae	Bird	46	1.17	25	37	Lane *et al.,* 2004
Cardinalis cardinalis	Passeriformes	Cardinalidae	Bird	41	1.81	29	42.6	Hinds & Calder, 1973
Cardinalis sinuatus	Passeriformes	Cardinalidae	Bird	32	1.41	30	42.8	Hinds & Calder, 1973
Carpodacus cassinii	Passeriformes	Fringillidae	Bird	27.4	1.22	22	37	Weather *et al.,* 1980
Carpodacus purpureus	Passeriformes	Fringillidae	Bird	34	3.06	25	35	Salt, 1952
Chordeiles minor	Caprimulgiformes	Caprimulgidae	Bird	72	1.59	30	39	Lasiewski *et al.,* 1970; Lasiewski & Dawson, 1964
Collocalia esculenta	Apodiformes	Apodidae	Bird	6.8	0.29	31.5	34	McNab & Bonaccorso, 1995
Collocalia vanikorensis	Apodiformes	Apodidae	Bird	11.6	0.43	30	34	McNab & Bonaccorso, 1995
Columba livia	Columbiformes	Columbidae	Bird	467	5.18	23	36.5	Calder & Schmidt-Nielsen, 1967
Coturnix coturnix	Galliformes	Phasianidae	Bird	97	3.21	16	34	Weathers, 1981; Hänßler, 1978
Coturnix japonica	Galliformes	Phasianidae	Bird	166	5.35	23	36	Ben-Hamo *et al.,* 2010
Ducula zoeae	Columbiformes	Columbidae	Bird	456.2	6.13	19	32	McNab, 2000
Emberiza chrysophrys	Passeriformes	Emberizidae	Bird	15.94	1.16	25	30	Jin-Song *et al.,* 2005
Emberiza pusilla	Passeriformes	Emberizidae	Bird	11.3	0.946	20	25	Jin-Song *et al.,* 2001
Emberiza rutila	Passeriformes	Emberizidae	Bird	15.3	1.22	25	32.5	Jin-Song *et al.,* 2001
Emberiza spodocephala	Passeriformes	Emberizidae	Bird	15.1	1.43	20	26	Ming *et al.,* 2005
Eremalauda dunni	Passeriformes	Alaudidae	Bird	20.6	1	31.5	41	Tieleman & Williams, 2002
Eremophila alpestris	Passeriformes	Alaudidae	Bird	26	1.185	26	35	Trost, 1972
Eurostopodus argus	Caprimulgiformes	Caprimulgidae	Bird	88	1.47	32	47	Dawson & Fisher, 1969
Fringilla montifringilla	Passeriformes	Fringillidae	Bird	21	1.38	25	30	Jin-Song *et al.,* 2004
Fulica atra	Gruiformes	Rallidae	Bird	387	8.47	20	34	Brent *et al.,* 1985
Geophaps plumifera	Columbiformes	Columbidae	Bird	89	1.52	33	39	Dawson & Bennett, 1973
Hemiphaga novaeseelandiae	Columbiformes	Columbidae	Bird	435.6	6.78	20	30	McNab, 2000
Icterus bullockii	Passeriformes	Icteridae	Bird	34	1.81	27	35	Rising, 1969
Icterus galbula	Passeriformes	Icteridae	Bird	34	1.81	27	35	Rising, 1969
Lagopus leucura	Galliformes	Phasianidae	Bird	326	8.5	6	38	Johnson, 1968
Lanius excubitor	Passeriformes	Laniidae	Bird	60	1.94	30	36	Ward & Pinshow, 1995; Degen *et al.,* 1992
Leipoa ocellata	Galliformes	Megapodiidae	Bird	1390	14.46	22	37	Booth, 1989
Lonchura fuscans	Passeriformes	Estrildidae	Bird	9.5	0.36	30	39	Weathers, 1977
Malurus cyaneus	Passeriformes	Maluridae	Bird	8.2	0.52	26	35	Lill *et al*., 2006
Megascops asio	Strigiformes	Strigidae	Bird	141.5	1.78	26	34	Ligon, 1969
Melopsittacus undulatus	Psittaciformes	Psittacidae	Bird	33.7	1.33	29	41	Weathers & Schoenbaechler, 1976
Micrathene whitneyi	Strigiformes	Strigidae	Bird	45	0.91	30	38	Ligon, 1968
Mirafra erythrocephala	Passeriformes	Alaudidae	Bird	27.3	1.5	27.9	35.1	Williams, 1999
Myiopsitta monachus	Psittaciformes	Psittacidae	Bird	80.4	1.89	24.5	38.5	Weathers & Caccamise, 1975
Passer domesticus	Passeriformes	Passeridae	Bird	23	1.32	20	35	Hudson & Kimzey, 1966
Pica nuttalli	Passeriformes	Corvidae	Bird	151.9	5.28	13.5	33.5	Hayworth *et al.*, 1984
Pica pica	Passeriformes	Corvidae	Bird	158.9	4.31	21	32.5	Hayworth *et al*., 1984
Pterocles alchata	Pterocliformes	Pteroclididae	Bird	242	3.38	28.6	32.1	Hinsley *et al.,* 1993
Pterocles orientalis	Pterocliformes	Pteroclididae	Bird	386.4	7.01	27.7	35.9	Hinsley *et al.,* 1993
Sterna fuscata	Charadriiformes	Sternidae	Bird	176.75	2.88	20	35	MacMillen *et al.,* 1977; Flint & Nagy, 1984
Strix occidentalis	Strigiformes	Strigidae	Bird	571	9.63	17	25.2	Ganey *et al*., 1993
Syrmaticus ellioti	Galliformes	Phasianidae	Bird	388.25	10.56	24.5	31.6	Ying *et al*., 2011
Syrmaticus humiae	Galliformes	Phasianidae	Bird	398.83	16.19	23	29.2	Ying *et al*., 2011
Tadorna variegata	Anseriformes	Anatidae	Bird	1193.6	12.04	22	33	McNab, 2003
Thinocorus rumicivorus	Charadriiformes	Thinocoridae	Bird	55.5	1.12	33	38	Ehlers & Morton, 1982
Todus mexicanus	Coraciiformes	Todidae	Bird	6.3	0.41	29	32	Merola-Zwartjes, 2000
Tyto alba	Strigiformes	Tytonidae	Bird	533.2	6.46	22.5	32.5	Edwards, 1987
Zenaida macroura	Columbiformes	Columbidae	Bird	91.4	2.33	30	37.5	Hudson & Brush, 1964
Zosterops erythropleurus	Passeriformes	Zosteropidae	Bird	9.16	0.85	25	27.5	Jin-Song *et al.,* 2005
